# Noninvasive prenatal screening for cystic fibrosis using circulating trophoblasts: Detection of the 50 most common disease‐causing variants

**DOI:** 10.1002/pd.6276

**Published:** 2022-12-08

**Authors:** Line Dahl Jeppesen, Dorte Launholt Lildballe, Lotte Hatt, Jakob Hedegaard, Ripudaman Singh, Christian Liebst Frisk Toft, Palle Schelde, Anders Sune Pedersen, Michael Knudsen, Ida Vogel

**Affiliations:** ^1^ ARCEDI Vejle Denmark; ^2^ Center for Fetal Diagnostics, Department of Clinical Medicine Faculty of Health Aarhus University Aarhus Denmark; ^3^ Department of Molecular Medicine Aarhus University Hospital Aarhus Denmark; ^4^ Department of Molecular Diagnostics Aalborg University Hospital Aalborg Denmark; ^5^ Center for Preimplantation Genetic Testing Aalborg University Hospital Aalborg Denmark; ^6^ Department of Clinical Genetics Aarhus University Hospital Aarhus Denmark

## Abstract

**Objectives:**

Cystic fibrosis (CF) is one of the most common severe autosomal recessive disorders. Prenatal or preconception CF screening is offered in some countries. A maternal blood sample in early pregnancy can provide circulating trophoblasts and offers a DNA source for genetic analysis of both the mother and the fetus. This study aimed to develop a cell‐based noninvasive prenatal test (NIPT) to screen for the 50 most common CF variants.

**Methods:**

Blood samples were collected from 30 pregnancies undergoing invasive diagnostics and circulating trophoblasts were harvested in 27. Cystic fibrosis testing was conducted using two different methods: by fragment length analysis and by our newly developed NGS‐based CF analysis.

**Results:**

In all 27 cases, cell‐based NIPT provided a result using both methods in agreement with the invasive test result.

**Conclusion:**

This study shows that cell‐based NIPT for CF screening provides a reliable result without the need for partner‐ and proband samples.

## Key points

**What's already known about this topic?**

- Many pregnant women are positive towards prenatal screening for Cystic fibrosis (CF).
- The only current pregnancy marker is echogenic bowel in the second trimester, but this test has low sensitivity and specificity.

**What does this study add?**

- Prenatal screening for CF can be done using circulating trophoblasts isolated from maternal blood early in pregnancy
- Circulating trophoblasts can provide screening for CF as a simple single‐visit setup without the need for a paternal sample.

## 1 INTRODUCTION

Cystic fibrosis (CF) is one of the most common recessive disorders. It is a multiple organ system disorder with onset in early childhood causing reduced life expectancy.^1^ The disease is caused by variants in the CF Transmembrane Conductance Regulator (*CFTR*) gene. More than 2000 different variants have been identified, and some are much more prevalent than others.^2^ In Denmark, approximately 3% of the population are carriers of a disease causing *CFTR*‐variant, the most common being F508del (*CFTR* c.1521_1523del), which is present in 96% of the Danish CF population.^3^, ^4^

In 2005, the American College of Obstetrics and Gynecology recommended to implement a prenatal or preconception CF carrier screening program.^5^, ^6^ In Denmark, however, such a screening program is not available, although it has been subject to discussion.^7^ Thus, a CF preconception carrier screening is only available for individuals with a family health history of CF. Verified carrier couples can opt for tax‐financed in vitro fertilization (IVF) and preimplantation genetic testing (PGT) or they can choose prenatal diagnosis for CF by chorionic villous sampling (CVS) or amniocentesis. Since 2016, newborn screening for CF has been implemented to ensure early intervention and treatment. In Denmark, 1–2 asymptomatic newborns are diagnosed with CF every month.^8^

‐Prenatal diagnosis traditionally relies on invasive sampling. Although studies indicate that procedure‐related risk of miscarriage is negligible,^9^, ^10^ the procedure is associated with discomfort. Invasive procedures are thus not adequate as a first‐tier screening and noninvasive solutions are warranted. In the second‐trimester ultrasound scan, findings of fetal echogenic bowel are used as markers for CF, although sensitivity and specificity are very low.^11^

The preference for noninvasive prenatal diagnostics for CF has been investigated in a study by Hill et al.^12^ The authors found that procedure‐related risk of miscarriage, test accuracy and the opportunity for early testing were the key attributes for accepting a noninvasive prenatal test (NIPT). The Danish couples in particular wished for a test without the risk of miscarriage.^13^ These findings were echoed in a questionnaire study among Danish PGT users, where 55% chose CVS to confirm transfer of an unaffected embryo. Nine out of 10 expressed a wish for a confirmatory test of the pregnancy if a noninvasive alternative was available.^14^ Therefore, efforts have been made to develop NIPT options for CF using either fetal cell‐free DNA or circulating trophoblasts in maternal blood.^15^, ^16^, ^17^

The procedure for NIPT using fetal cell‐free DNA was initially based on detection or exclusion of the paternal variant allele in maternal plasma.^18^ This approach is only useful if the parents carry different CF variants and is therefore not applicable for CF screening in a population with high mutation homogeneity such as the Danish. Moreover, detection of the paternal variant allele would require invasive testing to determine the fetal status for the maternal variant allele. Instead, an indirect testing strategy based on relative haplotype dosage analysis has been developed.^19^ This method was implemented as a clinical noninvasive diagnostic service in the UK in 2016 for couples where both parents are confirmed CF carriers and DNA is available from an affected proband or an unaffected child.^15^ This approach is thus not adequate for a population‐based prenatal CF screening program.

Intact circulating trophoblasts can be isolated consistently around gestational age (GA) 10–14 weeks and constitute the backbone in cell‐based NIPT. This allows the extraction of placental DNA without the background of maternal DNA.^20^ Circulating trophoblasts may therefore be useful for prenatal screening purposes, as direct variant analyses can be performed without the need for paternal‐ and proband samples.^16^, ^17^, ^21^, ^22^, ^23^

This aim of this study was to establish cell‐based NIPT as a screening tool for the 50 most common pathogenic *CFTR* variants. The analysis was conducted by direct variant analysis using either fragment length analysis or next‐generation sequencing (NGS) of circulating trophoblasts and test results were compared to results from invasive tests.

## 2 METHODS

### 2.1 Participant inclusion

Two groups of pregnant women undergoing invasive testing were included.

In group 1 (validation group), eight women (cases 1–8) with a risk of carrying a fetus with CF due to parental CF carrier status or family history (Table 1) were recruited at Department of Clinical Genetics, Aarhus University Hospital, Denmark.

**TABLE 1:** Summary of case characteristics and prenatal diagnosis for the validation group (group 1) containing pregnancy cases tested for Cystic fibrosis (CF) in the clinical setting

Case‐ID	Clinical indication	*CFTR* variant	Gestational age at blood sampling	Number of candidate cells for WGA and STR analysis	Number of fetal cells for cell‐based NIPT for *CFTR* variants	Cell‐based NIPT result for *CFTR* variants	Concordance between cell‐based NIPT and CVS results
1	Prior CF screen negative	None	13 + 2	5	4	Normal	Yes
2	Mother is a known carrier	F508del	12 + 4	6	5	F508del heterozygote	NA
3	One parent is a known carrier	F508del	11 + 4	8	1	Normal	Yes
4	Both parents are known carriers	F508del	11 + 3	11	4	F508del heterozygote	Yes
5	One parent is a known carrier	F508del	13 + 4	10	4	Normal	Yes
6	Both parents are known carriers	F508del, R334W	10 + 4	10	5	R334W heterozygote	Yes
7	Both parents are known carriers	F508del	9 + 5	10	1	Normal	Yes
8	Both parents are known carriers, child with CF	F508del	10 + 4	17	0	No result	‐

In group 2 (low‐risk group), 22 consecutive blood samples were collected between August and November 2021. This group included women (CF‐1 to CF‐22) opting for CVS at Department of Obstetrics and Gynecology, Aarhus University Hospital, Denmark, for indications other than CF (Table 2). Genetic counseling and follow‐up tests on the CVS material were offered at Department of Clinical Genetics, Aarhus University Hospital, if a variant was detected either in the pregnant women or by cell‐based NIPT.

**TABLE 2:** Summery of case characteristics and cell‐based noninvasive prenatal test (NIPT) results of 22 consecutive pregnancy cases (group 2) recruited for prenatal Cystic fibrosis (CF) screening when they opted for invasive sampling with an indication other than CF

Case‐ID	Gestational age at blood sampling	Number of candidate cells for WGA and STR analysis	Number of fetal cells for cell‐based NIPT for *CFTR* variants	Cell‐based NIPT result for *CFTR* variants	Concordance between cell‐based NIPT and CVS results
CF‐1	13 + 2	7	4	Normal	Yes
CF‐2	12 + 6	10	9	Normal	Yes
CF‐3	10 + 5	10	0	‐	‐
CF‐4	13 + 3	10	10	Normal	Yes
CF‐5	13 + 0	4	1	Normal	Yes
CF‐6	13 + 4	10	5	Normal	Yes
CF‐7	13 + 2	9	3	Normal	Yes
CF‐8	12 + 3^a^	10	1	Normal	Yes
CF‐9	12 + 6	10	7	Normal	Yes
CF‐10	12 + 5	10	8	Normal	Yes
CF‐11	11 + 1^a^	9	5	Normal	Yes
CF‐12	13 + 1^a^	4	0	‐	‐
CF‐13	10 + 4	10	4	Normal	Yes
CF‐14	9 + 4	10	6	Maternal carrier of F508del, fetus normal	Yes
CF‐15	12 + 1	10	6	Normal	Yes
CF‐16	12 + 3	8	5	Normal	Yes
CF‐17	13 + 6^a^	10	2	Normal	Yes
CF‐18	11 + 2	10	6	Normal	Yes
CF‐19	10 + 2	10	8	Normal	Yes
CF‐20	13 + 3	10	5	Maternal carrier of c.3718‐2477C > T, fetus normal	Yes
CF‐21	9 + 2	10	4	Normal	Yes
CF‐22	14 + 0	5	1	Normal	Yes

^a^ Indicates blood samples that have been collected after invasive sampling.

All (*N* = 30) included women donated a blood sample at GA 10–14 weeks; in 26 cases, blood collection was prior to CVS and 4 were taken after CVS.

The study was approved by Central Denmark Region Committee on Health Research Ethics (69,335, 72,586 and 79,316) and all participants gave informed consent prior to blood sampling after being informed of the project orally and in writing.

### 2.2 Blood processing and identification of circulating trophoblasts

Thirty ml of blood was drawn and processed as previously described.^24^, ^25^ In brief, three Cell‐Free DNA BCT tubes (Streck laboratories, USA) with whole blood were centrifuged and plasma was carefully removed from the cell pellet. This was followed by red blood cell lysis and paraformaldehyde‐fixation and permeabilization of nucleated cells. Next, enrichment by magnetic‐activated cell sorting was conducted using a LS column (MACS, Miltenyi Biotec, Germany) with CD105 and CD141 antibody conjugated microbeads. The enriched cell population was stained with fluorophore‐conjugated antibodies targeting a cocktail of cytokeratin (CK) antibodies, CD14 and CD45. Maternal genomic DNA (gDNA) was extracted using Maxwell® RSC Whole Blood DNA Kit (Promega, USA) following the manufacturer's protocol. In case 4 (group 1), only 20 ml of blood was drawn and for case 5 (group 1), 50 ml of blood. Candidate cells were isolated using a CK‐positive, CD14/CD45‐negative gate for single cell sorting using a BD fluorescens activated cell sorting (FACS)™Melody Cell Sorter (BD Biosciences, USA). This was followed by whole genome amplification (WGA) using PicoPLEX® Single Cell WGA Kit v3 (Takara Bio, USA) applied to up to 10 candidate cells per sample. GlobalFiler™ polymerase chain reaction (PCR) Amplification Kit (Thermo Fisher Scientific, USA) was applied to generate a Short Tandem Repeat DNA profile of candidate cells in order to determine the cell origin. Comparison with the maternal DNA profile allowed identification of trophoblasts based on the presence of paternally inherited alleles as previously described.^26^ If no cells of fetal origin were identified among the first 10 candidate cells, the remaining cells were analyzed accordingly.

### 2.3 Detecting common disease‐causing variants in Cystic Fibrosis Transmembrane Conductance Regulator

All samples in group 1, where trophoblasts were successfully harvested (*N* = 7), and all maternal (*N* = 22) and trophoblast WGA‐DNA samples (*N* = 20) from group 2 were subjected to CF analysis using two different methods: (i) Amplification Refractory Mutation System PCR (ARMS‐PCR) and fragment length analysis and (ii) NGS‐based CF analysis:

### 2.4 Amplification Refractory Mutation System PCR and fragment length analysis

The WGA products from single cells of fetal origin were diluted to 5 ng/μL and pooled by equal volume within cases. The pooled WGA‐DNA (and maternal gDNA) was analyzed using the Elucigene CFEU2v1 (Elucigene, UK) kit following manufacturer's instructions for detection of the 50 most common disease‐causing variants in *CFTR* by ARMS‐PCR. Fragment length analysis was performed using an Applied Biosystems 3500 Genetic Analyzer (Thermo Fisher Scientific, USA). Data was analyzed using GeneMapper™ 5 Software (Thermo Fisher Scientific, USA) using the Elucigene CFEU2v1 bins and panels. In the figures, the nomenclature for *CFTR* variants used in the “Elucigene CF‐EU2v1 Guide to Interpretation” was adopted (see supplementary Table 1). Thus, disease‐causing variant alleles are described as mutant alleles (e.g. F508del M) and normal alleles as wild‐type alleles (e.g. F508del wildtype [WT]).

### 2.5 Next‐generation sequencing‐based Cystic fibrosis analysis

NimaGen CFTR‐HS kit (version 0.3) was designed in collaboration with the manufacturer (NimaGen, The Netherlands). The analysis was performed following manufacturer's protocol: 20–80 ng maternal gDNA or pooled fetal WGA‐DNA was input for Reverse Complement‐PCR, where sample‐specific indexes and P5/P7 sequences (NimaGen) were added to create an NGS‐library for Illumina‐based sequencing with a mean insert size of 250 bp. Following equal volume pooling of the libraries and AMPure XP bead purification (Beckman Coulter), the libraries were sequenced on a MiniSeq (Illumina) in a 1x100 bp rapid run. Bcl2fastq and demultiplexing were performed using Local Run Manager (Illumina). The FASTQ files were aligned to the hg38 reference genome without alternate contigs^27^ using bwa mem (version 0.7.17) and the resulting BAM and VCF files were explored in Integrative Genomics Viewer (Broad Institute, UC San Diego).

The bioinformatic pipeline used SnpAhoy 0.5.2^28^ for calling the genotypes on 48 CFTR‐related SNP‐positions. SNP‐positions with genotype calls differing from the hg38 reference genome were reported as possible variants. The common 3‐base deletion F508del was detected using Freebayes 1.3.6^29^, ^30^ while the 21kb deletion CFTRdele2,3 was detected using Delly 0.9.1.^31^, ^32^ Filtering of the vcf‐file from Freebayes was done using VCFtools 0.1.16.^33^, ^34^

### 2.6 Allelic drop‐out rate

For the cell‐based NIPT results, the allelic drop‐out (ADO, i.e. loss of signal from one allele due to insufficient PCR amplification) rates were calculated by the number of observed alleles divided by the number of expected peaks for each sample: $ADO\;=\;1\;-\;\frac{Alleles\;observed}{Alleles\;expected}$.

For the ARMS‐PCR and fragment length analysis (Elucigene CFEU2v1), the ADO rate was calculated for the detection of variant alleles and the normal F508del allele (Elucigene CFEU2v1 reagent mix A), as well as the detection of the normal alleles (Elucigene CFEU2v1 reagent mix B, Supplementary Table 1). For all 27 cell‐based NIPT results from groups 1 and 2, all expected variant and normal F508del alleles were observed (*N* = 30), translating to a 0% ADO rate. In total, 1258 peaks out of 1330 expected normal and variant alleles were observed, translating to an ADO rate of 5.4% (CI_95_: 2.5%–8.4%, median: 2.0%, Supplementary Figure 1). For normal allele peaks <300 base pairs (peak no. 1–23 in CFEU2v1 reagent mix B, see Supplementary Table 1) and variant alleles (*N* = 3), the ADO rate was 0%.

For the NGS‐based CF analysis, the single nucleotide polymorphism (SNP) coverage depth was used to determine the ADO rate. The NimaGen CFTR‐HS includes 29 amplicons, covering the 50 disease‐causing variants of the Elucigene CFEU2v1 kit. Amplicon reads for CFTR‐HS‐Ampl‐26 are only generated if a CFTRdele2,3 (21kb deletion, c.54‐5940_273 + 10250del21080) variant allele is present. See Supplementary Table 1 for details. For all other amplicons, SNP‐coverage depth <300 reads was defined as SNP position ADO, allowing an SNP coverage depth of 30X with a minor allele frequency of 10%, which may occur due to WGA amplification bias. For all 27 samples, the average SNP ADO rate was 1.2% (CI_95_: 0.3%–2.2%, median: 0.0%, Supplementary Figure 2).

### 2.7 Cystic fibrosis testing on chorionic villous sampling

Pregnant women in group 1 had CVS for CF testing performed at regional hospitals of Central Denmark Region, Aarhus University Hospital or Aalborg University Hospital. For group 2, women were invited to participate in this study if they were offered CVS at Aarhus University Hospital for other reasons than CF; typically increased risk at the combined first‐trimester risk assessment. Chorionic villous sampling DNA was extracted at the Department of Clinical Genetics, Aarhus University Hospital, and analyzed using Elucigene CFEU2v1 in a clinical setting (group 1) or in a research setting (group 2).

## 3 RESULTS

**FIGURE 1:**
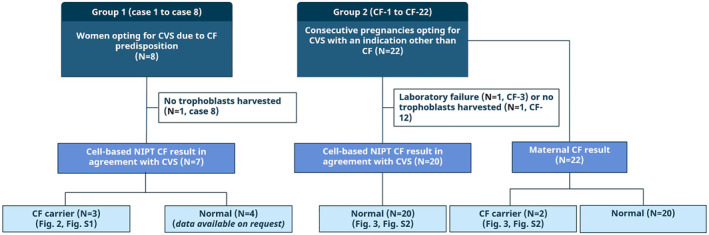
The flow of patients and samples. Blood was collected from two groups of pregnant women receiving invasive sampling. In group 1, the pregnancies were included when they opted for invasive sampling for Cystic fibrosis (CF) due to the couple's carrier status or family history. This group was the validation group. In group 2, *N* = 22 pregnant women were included consecutively when they opted for invasive sampling with an indication other than CF. This group simulated a prenatal screening program with a CF risk corresponding to the background population. The text describes the outcome for cell‐based noninvasive prenatal test (NIPT) for both groups as well as the maternal CF result in group 2. Abbreviations: CF, cystic fibrosis; CVS, chorionic villous sampling.

Flow of patients and samples is illustrated in Figure 1.

### 3.1 Group 1: Validation study with women opting for chorionic villous sampling for Cystic fibrosis

Cell‐based NIPT for CF was performed in eight pregnancies (GA 10–14) referred for prenatal CF diagnostics. Table 1 summarizes the results of cell‐based NIPT and invasive testing. In seven of these cases, a cell‐based NIPT result was successfully obtained and in six cases showing a fetal CF status in agreement with the CVS result. In case 2, the CVS result was not obtained. The consistency between the results of cell‐based NIPT and CVS accounts for both the ARMS‐PCR and fragment length‐ and the NGS‐based CF analysis. In one case (case 8), no fetal cells were harvested from the blood sample and a redraw was not an option.

**FIGURE 2:**
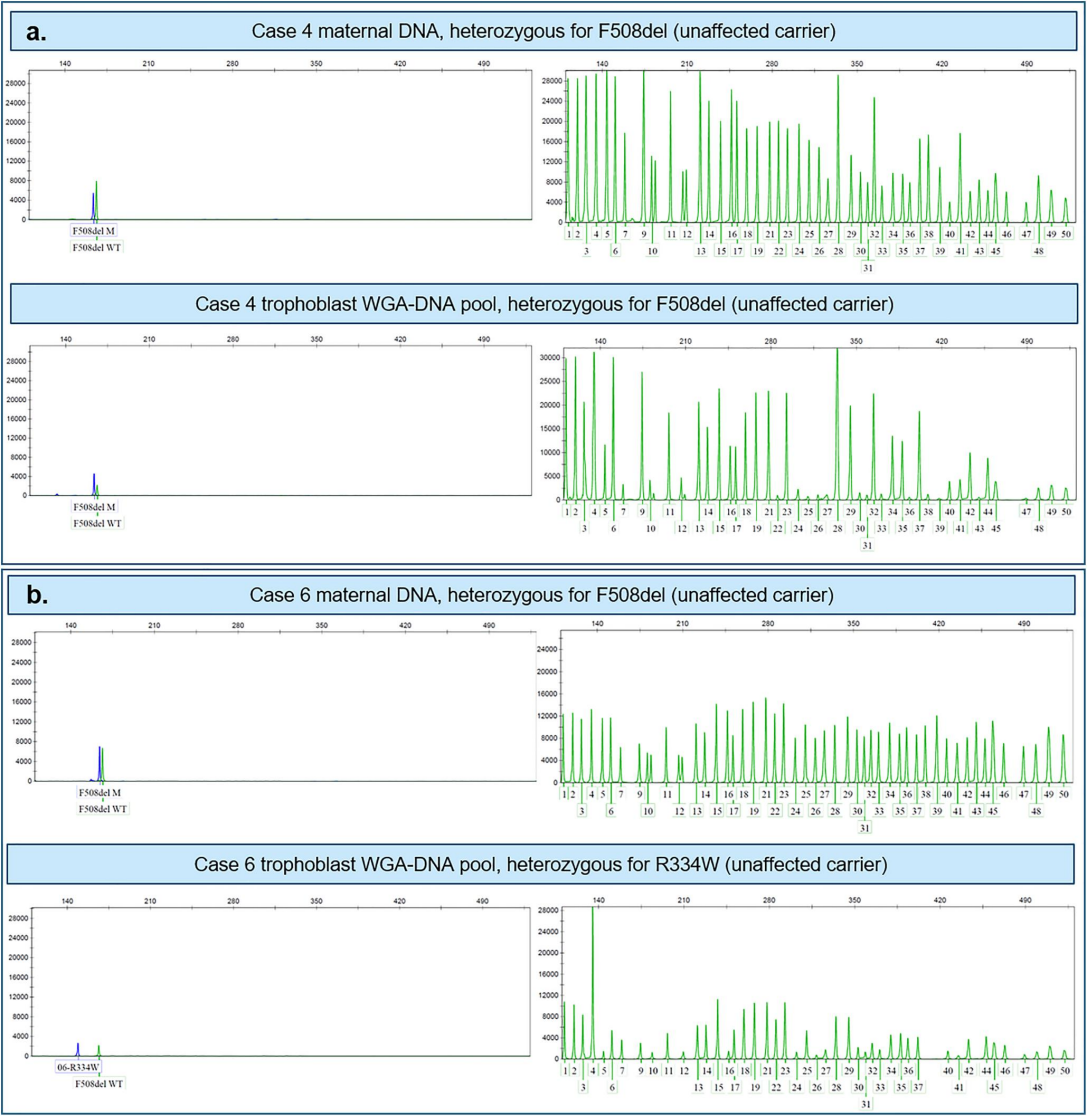
CF test results for maternal DNA and trophoblast WGA‐DNA pools for case 4 and case 6. The fragment length analysis results to the left show the variant (mutant, M) alleles represented by blue peaks and the normal (wildtype) F508del allele represented by a green peak. The fragment length analysis results to the right show the normal alleles and detection of a green peak indicates the heterozygote status of the individual, while the absence of the green peak indicates the homozygote status of the individual. Peaks 10 and 12 represent F508del, which present as split peaks separated by 3 bp if both a normal allele and a variant allele are present. (A) In case 4, both the pregnant woman and the fetus are heterozygote for F508del indicated by the F508del M and WT alleles, as well as 10 and 12 split peaks in the fragment length analysis result to the right. (B) In case 6, the pregnant woman is a heterozygote carrier of F508del. The fetus has inherited the paternal R334W variant allele and the normal F508del allele, indicating that the fetus is an unaffected carrier of Cystic fibrosis (CF). Abbreviations: CF, cystic fibrosis; M, mutant, WT, wildtype; WGA‐DNA, whole genome amplified DNA.

As can be seen from Table 1, *CFTR* variants in the heterozygote form were detected in the fetus of cases 2, 4 and 6. Figure 2A shows the results for case 4, where the mother and the fetus are both heterozygote carriers of the pathogenic variant commonly known as F508del (*CFTR* c.1521_1523del). The first panel row shows the result for maternal blood DNA and the second panel shows the result from the trophoblasts retrieved from the same blood sample. Maternal and fetal samples both present with a variant allele and a normal allele. For details, see the figure legend.

Figure 2B shows the finding of a paternal variant allele, R334W (*CFTR* c.1000C > T), in the fetus, as well as the normal F508del allele (WT). Importantly, the maternal F508del variant allele is not present in the fetus; thus, the child is an unaffected CF carrier.

All fetal WGA‐DNA samples from group 1 (*N* = 7) were analyzed by NGS to establish a proof‐of‐principle. Supplementary Figure 3 shows the NGS‐based CF analysis result for cases 4 and 6. For all cases, the results of NGS analysis were in concordance with the CVS result.

### 3.2 Group 2: Prenatal Cystic fibrosis screening of consecutive pregnancies opting for chorionic villous sampling for an indication other than Cystic fibrosis

In the second part of the study, 22 consecutive samples (CF‐1 to CF‐22) were collected from pregnant women opting for CVS for indications other than CF. The case characteristics are summarized in Table 2. For 20 samples, a cell‐based NIPT result was obtained by both ARMS‐PCR and fragment length and NGS‐based CF analysis; these results were identical with those of the invasive samples. Two cases failed to generate a cell‐based NIPT result: One sample (CF‐3) failed due to a technical error during FACS, and from the other sample (CF‐12), no trophoblasts were harvested.

**FIGURE 3:**
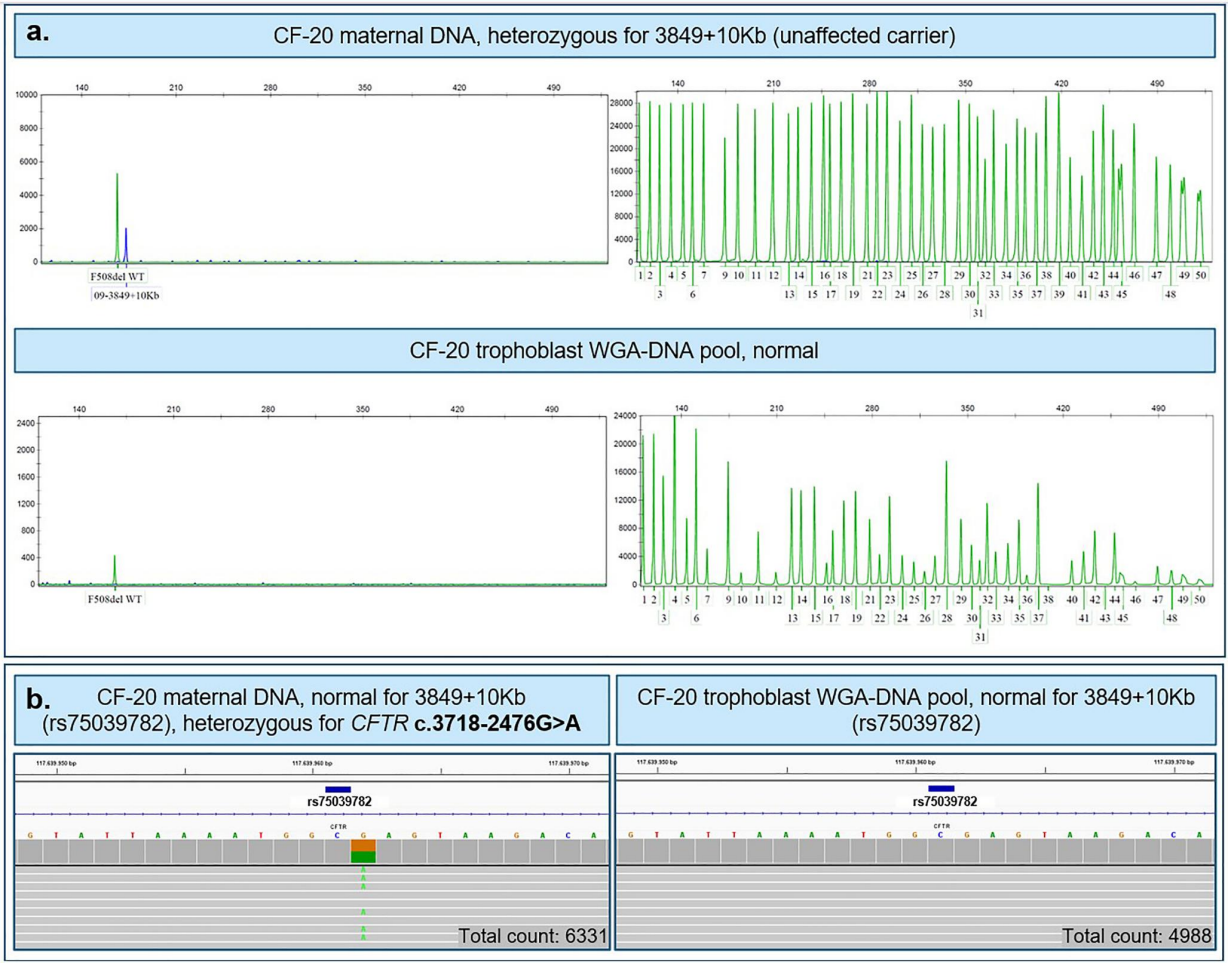
CF test result for maternal DNA and trophoblast WGA‐DNA pools for CF‐20 using (A) Amplification Refractory Mutation System polymerase chain reaction (ARMS‐PCR) and fragment length analysis and (B) next‐generation‐sequencing. (A) The maternal Cystic fibrosis (CF) test result presents with a blue variant peak 09–3849 + 10Kb indicating that the pregnant woman is a heterozygote carrier of Cystic Fibrosis Transmembrane Conductance Regulator (CFTR) 3849 + 10kbC > T (c.3718‐2477C > T, dbSNP: rs75039782). The trophoblast WGA‐DNA showed a normal CF test result, indicating that the fetus is unaffected. Panel (B) shows the sequencing analysis of the variant, rs75039782, and the base position is marked in dark blue bar. The grey bars represent sequences that do not vary from the hg38 reference genome, displayed by the colored base sequence. In the maternal test result, an single nucleotide variant (SNV), CFTR c.3718‐2476 G > A, was detected 1 bp downstream for the variant of interest. This SNV is a likely benign intron variant. Thus, the ARMS‐PCR result was due to an SNV in the primer binding site, resulting in a false‐positive result. The CF‐20 trophoblast WGA‐DNA sequencing result presented with a normal CF test result. Total counts for the specific amplicon are specified in the lower right corner. Abbreviations: ARMS‐PCR, Amplification Refractory Mutation System polymerase chain reaction; bp, base pair; CF, cystic fibrosis; SNV, single nucleotide variant; WGA‐DNA, whole genome amplified DNA.

In two maternal samples (CF‐14 and CF‐20), a CF variant allele was detected. In the first sample, the pregnant woman carried an F508del variant allele, while the cell‐based NIPT result showed a normal CF profile in the fetus, confirmed by CVS (Supplementary Figure 4). The second sample, CF‐20, was more complicated (see Figure 3). The maternal fragment length analysis indicated that she was a heterozygous (carrier) of *CFTR* 3849 + 10kbC > T (*CFTR* c.3718‐2477C > T). The cell‐based NIPT result presented with a normal CF profile in agreement with the CVS result (Figure 3A). The pregnant woman was informed about the result, and the fragment length analysis in the clinical setting confirmed the result. However, the NGS‐based CF analysis deviated (Figure 3B) as a single nucleotide variant (SNV) in the maternal sequence was found 1bp downstream from the reported variant. This SNV was the *CFTR* c.3718‐2476G > A intron variant categorized as likely benign (https://www.ncbi.nlm.nih.gov/clinvar/RCV000870174/, Figure 3). This deviating result was confirmed by Sanger sequencing at the Department of Clinical Genetics, Aarhus University Hospital and communicated to the pregnant couple. The fragment length analysis manufacturer (Elucigene, Yourgene Health) will include this observation in the revised user instructions.

### 3.3 Next‐generation sequencing‐based Cystic fibrosis analysis

An NGS‐based CF analysis was performed using Nimagen CFTR‐HS kit for all 27 fetal WGA‐DNA samples. In brief, the MiniSeq run generated a total yield of 1.88 G, with 95.64% of data considered high quality (% >=Q30). The SNP coverage depth for each variant is shown in Supplementary Figure 5, including the total mean coverage depth for all 27 fetal WGA‐DNA samples.

Supplementary Figure 3 shows the NGS‐based CF screening result for case 4, where both the mother and the fetus were heterozygote carriers of an F508del variant allele. For case 6, the NGS‐based CF screening result for the F508del variant and the R334W variant (*CFTR* c.1000C > T) is shown in Supplementary Figure 3.

## 4 DISCUSSION

The presented data showed that noninvasive testing using circulating trophoblasts can determine fetal CF status. A cell‐based NIPT result was obtained in 27 cases; seven cases at a high risk of CF, and 20 consecutive pregnancies undergoing invasive diagnostics on indications other than CF. In all cases, cell‐based NIPT provided an accurate result in agreement with the invasive diagnostics. This reveals the potential of cell‐based NIPT to provide an accurate and timely prenatal screening for CF without the need for partner or proband samples.

Is cell‐based NIPT then an alternative to invasive testing? We found four of seven high‐risk cases, where the cell‐based NIPT showed a normal unaffected CF test result. The remaining three high‐risk cases showed the presence of both a normal allele and a variant allele, indicating that the fetuses were unaffected CF carriers. These three cases reflect clinical situations where invasive testing could be avoided. However, we recognize that invasive confirmatory testing is needed in situations where allelic drop‐ssssout could influence interpretation of the result as cases with homozygous normal results when the parents carry different CF variants or homozygous abnormal results.

While awaiting larger validation studies, cell‐based NIPT for CF may be relevant: (i) when CF carrier couples abstain from prenatal testing due to the procedure‐related risk of miscarriage, (ii) if CF is suspected due to the presence of ultrasound fetal hyperechogenic bowel, and (iii) to confirm the transfer of an unaffected embryo following PGT.^35^

In the second part of this study, cell‐based NIPT for CF was performed in 22 consecutive pregnant women opting for CVS for reasons other than CF, simulating a prenatal screening setup. A cell‐based NIPT CF screening result was obtained in 20 of the 22 fetuses and they all showed a normal CF test result in agreement with the CVS results. In the two remaining pregnancies, a cell‐based NIPT result was not achieved, which is equivalent to a failure rate of 9% (CI: 1%–29%). For future large‐scale validation studies, the failure rate may be reduced by sampling an increased amount of blood (e.g. from 30 to 60 ml) or by a redraw only if a cell‐based NIPT result cannot be achieved based on the first sample. However, the residual risk of fetal CF was significantly lowered as the maternal result was normal. In two maternal results, the fragment length analysis for CF found a variant. In both cases, follow‐up analysis, genetic counseling and CF testing of the partner and relevant family members were offered. Overall, these results suggest that trophoblasts can be a DNA source in prenatal CF screening.

The DNA source for cell‐based NIPT is limited to the amount obtained from few harvested trophoblasts, and WGA is therefore a necessity to obtain sufficient DNA for genetic analysis. However, the WGA procedure leads to the risk of amplification of only one allele and lack of amplification of the other (ADO). If the nonamplified allele is the variant allele, this will cause a wrong diagnosis. Thus, ADO is a critical measure of the test performance. For the cell‐based NIPT results using both fragment length analysis and NGS (*N* = 27), the ADO rates were 5.4% (CI_95_: 2.5%–8.4%, median: 2.0) and 1.2% per sample (CI_95_: 0.3%–2.2%, median: 0.0%), respectively. These ADO rates are lower than what has previously been reported in similar studies using WGA‐DNA from circulating trophoblasts (27%–74%).^22^, ^23^ In this study, the initial material for the CF analysis was WGA‐DNA pooled from multiple trophoblasts, and this may explain the lower ADO rate. Thus, cell‐based NIPT for CF should preferably be based on WGA‐DNA from multiple trophoblasts as recommended by Vossaert et al.^36^ The technology used in the present study holds promise for retrieval of an adequate number of trophoblasts from most samples obtained in GA 10–14 weeks.^20^, ^37^ Thus, the cell‐based prenatal CF screening could potentially be accessible prior to the first‐trimester screening at 12 full weeks of gestation. Downstream analysis may vary according to volume and clinical preferences, and in the following, we will discuss our experiences with the clinically established fragment length analysis and the NGS‐based CF analysis developed for this purpose in our clinical samples.

In this study, we found that the NGS platform is just as useful as the fragment length analysis and may offer some potential benefits even though some barriers remain. In one woman, both the CF screening test and the clinical follow‐up analysis detected a less common variant (*CFTR* c.3718‐2477C > T) using fragment length analysis. Surprisingly, this result was not confirmed in the NGS analysis, which instead showed a neighboring SNP (*CFTR* c.3718‐2476G > A). This changed the clinical interpretation as this variant is interpreted as likely benign. SNPs in the primer annealing regions of other PCR variant allele amplicons may result in false‐positive results, which may become more predominant when used in population‐based screening, as compared to diagnostic procedures. Similarly, in the NGS‐based CF analysis, there is a potential for detection of variants of unknown significance, which can be a real burden for both health care professionals and the expectant parents. However, this barrier can be overcome by using a bioinformatic pipeline to call variants of interest. The high analysis cost for the NGS‐based CF analysis is a barrier to use this method instead of fragment length analysis when analyzing small sample sizes but would be overcome in a large‐scale screening setup. Thus, the advantages of the NGS platform includes correct identification of the SNPs, as in the case above, lower ADO rate (1.2% per sample vs. 5.4% in the fragment length analysis), bioinformatic variant calling and scalability for a screening setup.

In a recent study, Chang et al. presented a cell‐based NIPT analysis using circulating trophoblasts for targeted sequencing of a gene panel including 67 genes related to different monogenic diseases.^22^ The approach used both direct variant analysis and SNPs for haplotyping, requiring a blood sample from the partner and a proband. Another recent study by Zhuo et al. used a similar approach to detect pathogenic variants for Tay Sachs disease, CF and hemoglobinopathies, as well as to detect family‐specific pathogenic variants.^23^ Together, these studies support the feasibility for developing cell‐based NIPT for various monogenic disorders using NGS.

The economy for cell‐based NIPT CF screening at a national scale has not yet been addressed, but cost‐benefit analyses for genetic carrier screening in CF have previously been positive.^38^ Implementation of cell‐based CF screening will identify CF carriers, which will allow them to consider their reproductive options before conception. This is opposed to the current newborn screening program, which does not accurately identify CF carrier individuals.

## 5 CONCLUSION

Cell‐based NIPT provides an option for a prenatal CF screening program for the 50 most common disease‐causing variants. Importantly, this can be done early in pregnancy and without the need for a partner sample and with little inconvenience for the pregnant woman.

## CONFLICT OF INTEREST

Line Dahl Jeppesen, Lotte Hatt, Jakob Hedegaard, Ripudaman Singh, and PS are all employed by ARCEDI, a Danish biotech company that holds the patented technology for enrichment of circulating trophoblasts used in this study. Anders Sune Pedersen and Michael Knudsen are employed as consultants by ARCEDI. Dorte Launholt Lildballe, Christian Liebst Frisk Toft, and Ida Vogel have no conflicts of interest and do not receive any funding by ARCEDI.

## Supporting information

Supplementary Material

Supplementary Material

## Data Availability

Study data not presented in this article is available upon request directed to the corresponding author.
