# Supplementary material for: Noninvasive prenatal screening for cystic fibrosis using circulating trophoblasts: Detection of the 50 most common disease‐causing variants
Source: Prenat Diagn. 2022 Dec 8;43(1):3–13. doi: 10.1002/pd.6276 (PMC10107343; doi:10.1002/pd.6276)
Supplement: Supplementary file 1 — Supplementary Material [file PD-43-3-s001.docx]

# Supplementary

**Supplementary Table 1** Cystic Fibrosis variants characterization and details for the laboratory using the fragment length analysis (Elucigene CFEU2v1) and the NGS-based analysis (NimaGen CFTR-HS), respectively.

| **Position (hg38)** | **cDNA name** | **Elucigene CFEU2v1 Marker** | **Elucigene CFEu2v1 Marker (Peak No.)** | **RefSNP** | **NimaGen CFTR-HS amplicon** |
| --- | --- | --- | --- | --- | --- |
| 117498313 | c.54-5940_273+10250del21080 | CFTRdele2,3 | 41 |  | CFTR-HS-Ampl-26 (an amplicon is generated if the deletion is present) |
| 117509047 | c.178G>T | E60X | 44 | rs77284892 | CFTR-HS-Ampl-01 |
| 117509069 | c.200C>T | P67L | 42 | rs368505753 | CFTR-HS-Ampl-01 |
| 117509123 | c.254G>A | G85E | 35 | rs75961395 | CFTR-HS-Ampl-01.2 |
| 117509131 | c.262_263delTT | 394delTT | 34 | rs121908769 | CFTR-HS-Ampl-01.2 |
| 117530938 | c.313delA | 444delA | 27 | rs121908801 | CFTR-HS-Ampl-02 |
| 117530974 | c.349C>T | R117C | 31 | rs77834169 | CFTR-HS-Ampl-02 |
| 117530975 | c.350G>A | R117H | 30 | rs78655421 | CFTR-HS-Ampl-02 |
| 117530991 | c.366T>A | Y122X | 33 | rs79660178 | CFTR-HS-Ampl-02 |
| 117531115 | c.489+1G>T | 621+1G>T | 47 | rs78756941 | CFTR-HS-Ampl-03 |
| 117534366 | c.579+1G>T | 711+1G>T | 5 | rs77188391 | CFTR-HS-Ampl-04 |
| 117535285 | c.617T>G | L206W | 13 | rs121908752 | CFTR-HS-Ampl-05 |
| 117540178 | c.948delT | 1078delT | 11 | rs121908744 | CFTR-HS-Ampl-06a |
| 117540230 | c.1000C>T | R334W | 6 | rs121909011 | CFTR-HS-Ampl-06b |
| 117540270 | c.1040G>C, c.1040G>A | R347H, R347P | 1;2 | rs77932196 | CFTR-HS-Ampl-06b |
| 117548795 | c.1364C>A | A455E | 48 | rs74551128 | CFTR-HS-Ampl-07 |
| 117559590 | c.1519_1521delATC | I507del | 7 | rs1801178 | CFTR-HS-Ampl-08 |
| 117559592 | c.1521_1523delCTT | F508del | 8 | rs113993960 | CFTR-HS-Ampl-08 |
| 117559616 | c.1545_1546delTA | 1677delTA | 10 | rs121908776 | CFTR-HS-Ampl-08 |
| 117559629 | c.1558G>T | V520F | 12 | rs77646904 | CFTR-HS-Ampl-08 |
| 117587738 | c.1585-1G>A | 1717-1G>A | 29 | rs76713772 | CFTR-HS-Ampl-09.2 |
| 117587778 | c.1624G>T | G542X | 23 | rs113993959 | CFTR-HS-Ampl-09.1 |
| 117587800 | c.1647T>G | S549N | 21 | rs121908755 | CFTR-HS-Ampl-09.1 |
| 117587801 | c.1646G>A | S549R(T>G) | 20 | rs121909005 | CFTR-HS-Ampl-09.1 |
| 117587806 | c.1652G>A | G551D | 19 | rs75527207 | CFTR-HS-Ampl-09.1 |
| 117587811 | c.1657C>T | R553X | 18 | rs74597325 | CFTR-HS-Ampl-09.1 |
| 117587833 | c.1679G>C | R560T | 15 | rs80055610 | CFTR-HS-Ampl-09.1 |
| 117589467 | c.1680-886A>G | 1811+1.6kbA>G | 28 | rs397508266 | CFTR-HS-Ampl-10 |
| 117590440 | c.1766+1G>A | 1898+1G>A | 37 | rs121908748 | CFTR-HS-Ampl-12 |
| 117592179 | c.2012delT | 2143delT | 43 | rs121908812 | CFTR-HS-Ampl-13 |
| 117592219 | c.2052delA | 2184delA | 39 | rs121908746 | CFTR-HS-Ampl-13 |
| 117592382 | c.2215delG | 2347delG | 16 | rs397508353 | CFTR-HS-Ampl-14 |
| 117594976 | c.2538G>A | W846X | 38 | rs397508393 | CFTR-HS-Ampl-15 |
| 117602868 | c.2657+5G>A | 2789+5G>A | 3 | rs80224560 | CFTR-HS-Ampl-16 |
| 117603542 | c.2668C>T | Q890X | 17 | rs79633941 | CFTR-HS-Ampl-17 |
| 117606754 | c.2988+1G>A | 3120+1G>A | 4 | rs75096551 | CFTR-HS-Ampl-18 |
| 117611555 | c. 3140-26A>G | 3272-26A>G | 46 | rs76151804 | CFTR-HS-Ampl-19a |
| 117611637 | c.3196C>T | R1066C | 36 | rs78194216 | CFTR-HS-Ampl-19b |
| 117611717 | c.3276C>A | Y1092X(C>A) | 25 | rs121908761 | CFTR-HS-Ampl-20 |
| 117611743 | c.3302T>A | M1101K | 22 | rs36210737 | CFTR-HS-Ampl-20 |
| 117614699 | c.3454G>C | D1152H | 40 | rs75541969 | CFTR-HS-Ampl-21 |
| 117627525 | c.3472C>T | R1158X | 59 | rs79850223 | CFTR-HS-Ampl-22 |
| 117627537 | c.3484C>T | R1162X | 49 | rs1800120 | CFTR-HS-Ampl-22 |
| 117627581 | c.3528delC | 3659delC | 45 | rs121908747 | CFTR-HS-Ampl-22 |
| 117639961 | c.3718-2477C>T | 3849+10kbC>T | 9 | rs75039782 | CFTR-HS-Ampl-23 |
| 117642472 | c.3752G>A | S1251N | 26 | rs74503330 | CFTR-HS-Ampl-24a |
| 117642493 | c.3773dupT | 3905insT | 24 | rs796566397 | CFTR-HS-Ampl-24a |
| 117642566 | c.3846G>A | W1282X | 14 | rs77010898 | CFTR-HS-Ampl-24b |
| 117652877 | c.3909C>G | N1303K | 32 | rs80034486 | CFTR-HS-Ampl-25 |

**Supplementary Figure 1:** Allelic dropout rate for ARMS-PCR and fragment length analysis for all variant- and normal alleles for fetal WGA-DNA samples (N = 27)


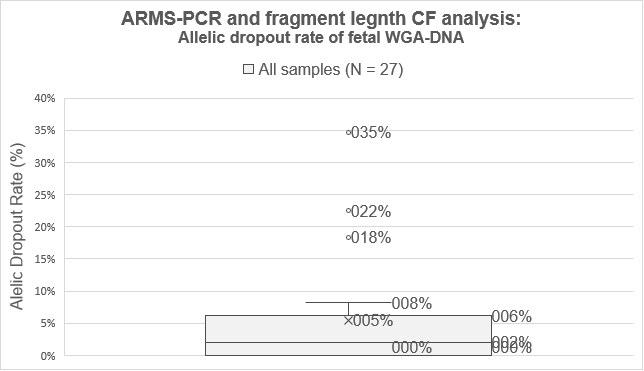


**Supplementary Figure 2:** Allelic dropout rate for fetal WGA-DNA samples (N = 27) for NGS-based cystic fibrosis with 29 amplicons covering the 50 most common cystic fibrosis variants.


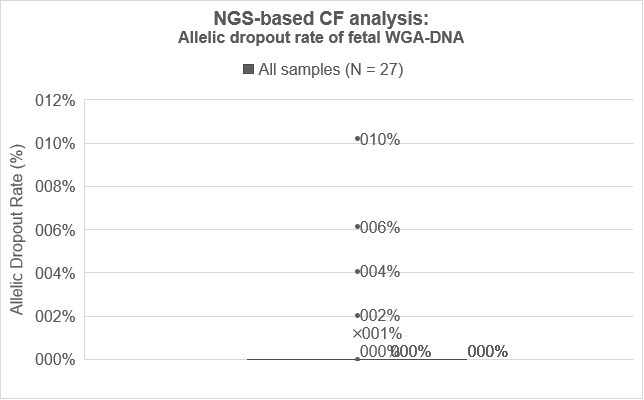


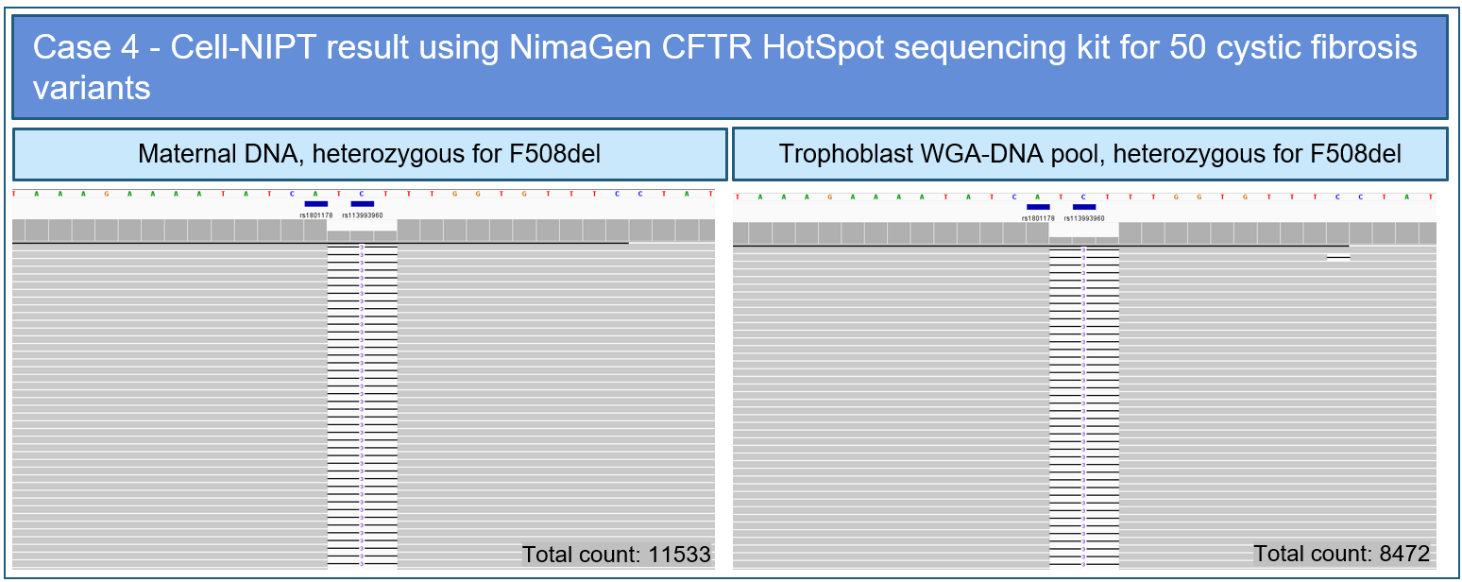

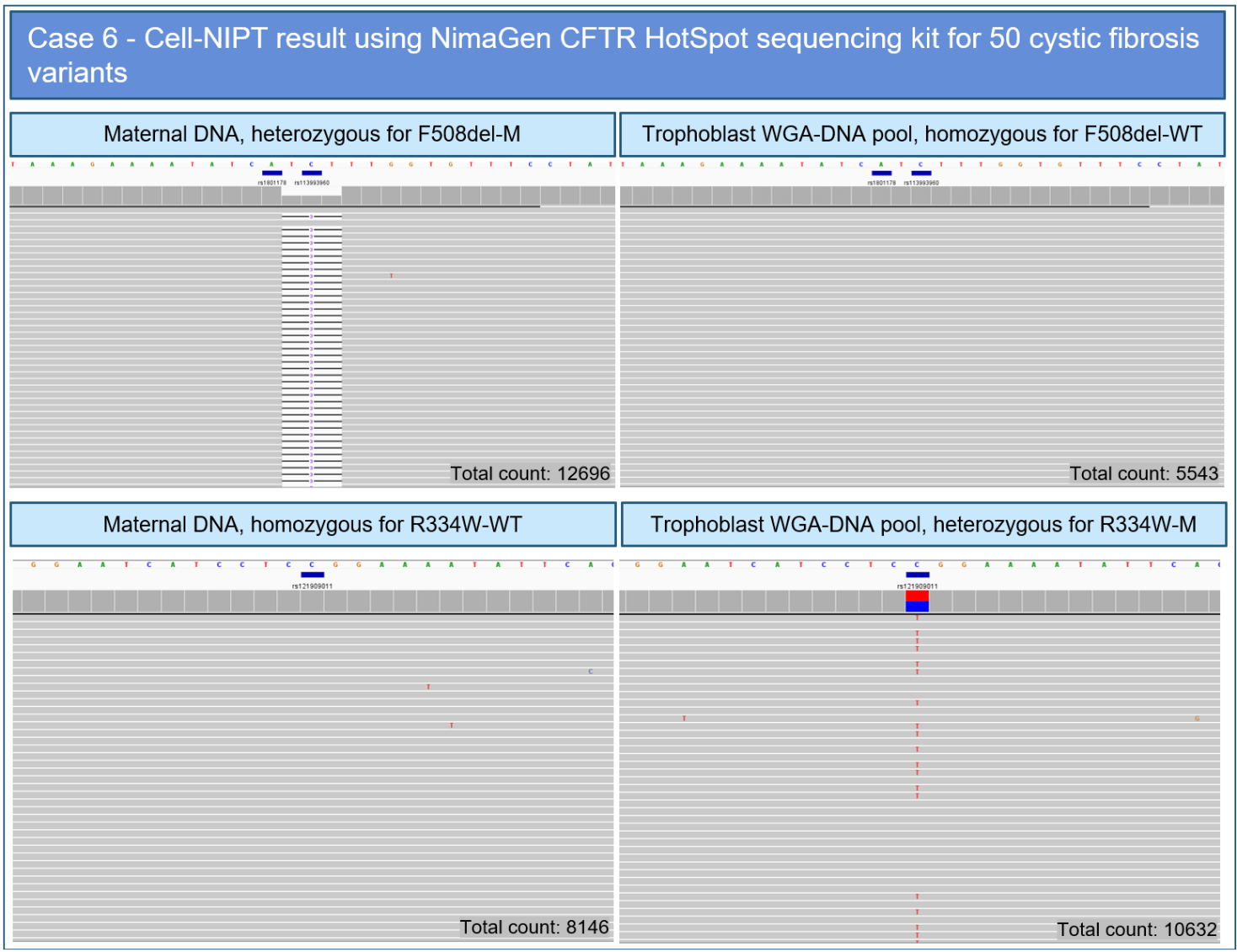


**Supplementary Figure 3**: **Cell-based NIPT result for case 4 and 6 using NimaGen CFTR HotSpot sequencing kit for 50 cystic fibrosis variants**. Pathogenic cystic fibrosis variants in the regions shown are marked by the blue bars and their rs identification number. The hg38 reference sequence is given in the top of the window in colored bases, Amplicons with sequence identical to the reference presents as grey horizontal bars. Total counts are specified in the right lower corner for the specific amplicon. In case 4, both the maternal DNA and the trophoblasts WGA-DNA test results were heterozygote for the three basepair deletion at rs113993960, which corresponds to CFTR F508del. In case 6, the pregnant woman was heterozygote for F508del, and her partner was heterozygote for R334W,c.1000C>T. The trophoblast WGA-DNA test result shows that the fetus is homozygote for the normal allele at rs113993960 (F508del), and has inherited the paternal variant identified as a C>T mutation at the position marked as rs121909011. This result indicates that the fetus is an unaffected CF carrier.


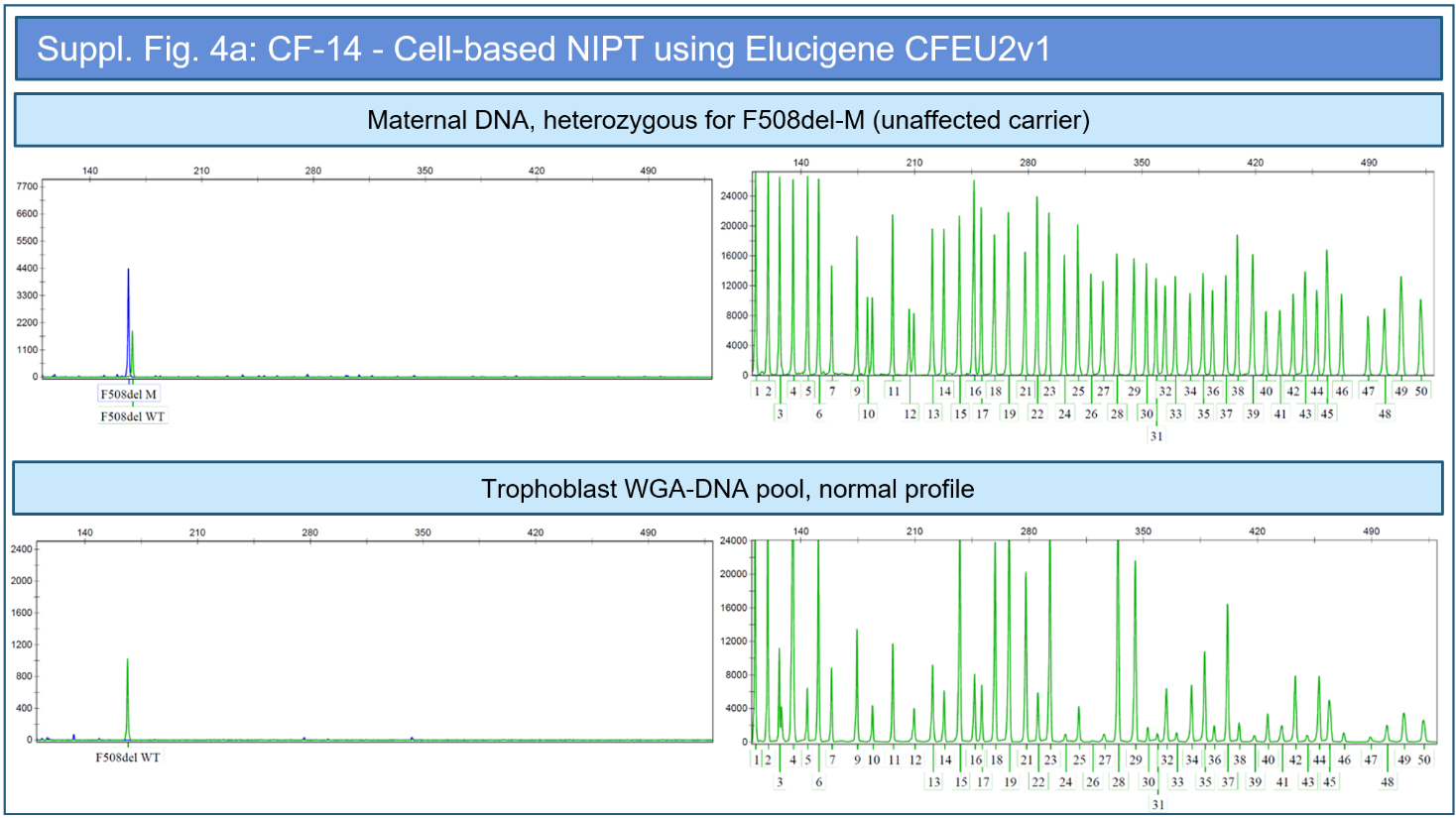

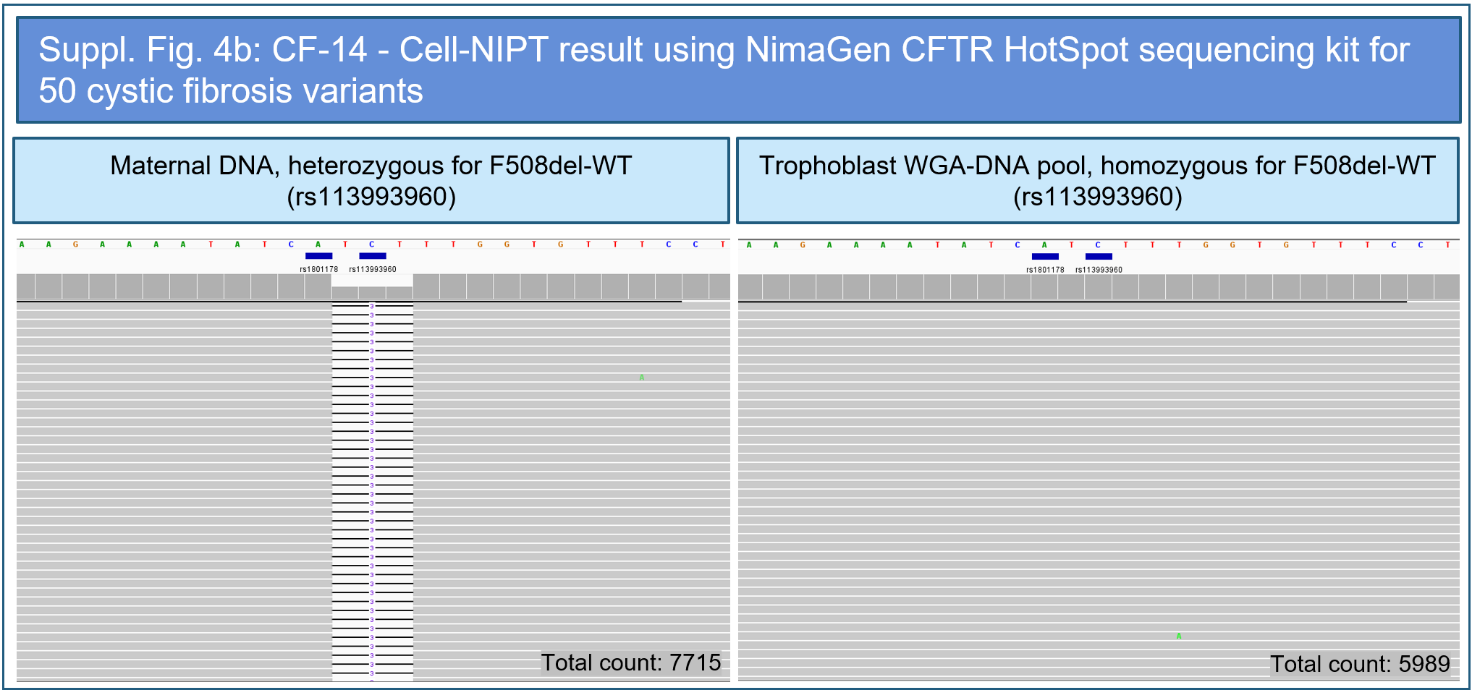


**Supplementary Figure 4: ARMS-PCR and fragment length analysis result for CF-14. Fig. 4a:** *The upper panel shows the result for maternal genomic DNA and the lower panel shows the cell-based NIPT result based on a pool of WGA-DNA amplified from 6 trophoblasts. The fragment length analysis result at the left shows the detection of variant alleles (mutant, M), indicated by blue peaks, and the normal (wildtype, WT) F508del allele represented by a green peak. The fragment length analysis result at the right shows the results the detection of the normal alleles, which is applied to identify a heterozygote individual following detection of a specific variant allele. For CF-14 maternal DNA, an F508del variant allele and -normal allele are identified, indicating that she is an unaffected carrier. The trophoblast WGA-DNA presents with a normal CF test result.* ***Fig. 4b:*** *The result for two of the fifty cystic fibrosis variants analyzed by next generation sequencing. The base position of the disease-causing cystic fibrosis variant is marked by the blue bars and identified by an rsID. The grey color represents sample reads identical with the hg38 reference genome sequence, which is displayed in colored bases in the top. Total counts for the specific amplicon are specified in the right lower corner. The maternal DNA represents with a three basepair deletion as the rs113993960, corresponding to the F508del variant which was also detected by the fragment analysis. The three basepair deletion is present in half of the amplicon reads, indicating that the pregnant woman is heterozygote for the F508del variant. The trophoblast WGA-DNA shows a sequence corresponding to the reference genome. This result is in concordance with the results of fragment length analysis, and the CF analysis of the invasive sample.*

**Supplementary Figure 5: see separate file**
